# Supplementary material for: The transcription factor GATA4 promotes myocardial regeneration in neonatal mice
Source: EMBO Mol Med. 2017 Jan 4;9(2):265–79. doi: 10.15252/emmm.201606602 (PMC5286367; doi:10.15252/emmm.201606602)
Supplement: Supplementary file 1 — Appendix [file EMMM-9-265-s001.pdf]

Appendix PDF

Appendix Table S1

Appendix Table S2

Appendix Table S3

Category	Gene	WT sham	KO sham	WT cryo	KO cryo
Cell cycle promoting	Ccna2	1	0.65±0.015**	0.73±0.056	0.37±0.07###
	Ccnb1	1	1.11±0.04	1.14±0.05	1.12±0.2
	Ccnd1	1	0.89±0.07	0.77±0.04	0.94±0.09
	Ccnd2	1	0.78±0.1	1.02±0.03	1.23±0.09
	Ccnd3	1	0.74±0.05	0.75±0.09	0.65±0.04
	Ccne1	1	0.63±0.06**	0.54±0.07	0.79±0.07
	Cdk4	1	0.58±0.06**	0.76±0.09	0.67±0.012
	E2f1	1	0.47±0.01*	0.68±0.08	0.549±0.02
	Cenpa	1	0.655±0.027**	0.8±0.025	0.51±0.0058###
	Zfp191	1	1.06±0.14	1.03±0.06	0.38±0.1##
	Cdc2 (cdk1)	1	0.59±0.07*	0.89±0.11	0.8±0.06
Cell cycle inhibiting	Rbl1 (p107)	1	0.94±0.11	0.86±0.13	0.47±0.05#
	Rab3gap1(p130)	1	0.98±0.17	1.1±0.06	0.522±0.04##
	Tsc22d1	1	1.84±0.62	1.22±0.13	2.00±0.31#
	Meis1	1	1.12±0.19	0.9±0.07	0.46±0.05##
Regenerative cytokines or receptors	Il13	1	1.25±0.22	1.1±0.075	0.65±0.096#
	Ctf1	1	1.23±0.3	0.93±0.49	0.878±0.15
	Igf1	1	0.91±0.046	N/A	N/A
	Igf2r	1	1.1±0.189	1.12±0.066	0.56±0.068##
	Vegfa	1	1.3±0.28	1.1±0.08	0.51±0.11#
	Fgf16	1	0.5±0.13	0.76±0.19	0.71±0.44
Anti-regenerative cytokines	Tgfb	1	1.02±0.06	0.933±0.19	0.87±0.08

Appendix Table S1: Relative regenerative gene expression (average ± SEM) in the heart of wild-type (WT) or cardiomyocyte specific *Gata4* knock-out (KO) mice (n=3/group) 7 days after cryoinjury (cryo) or sham surgery.*p<0.05, **p<0.01, ***p<0.001 vs WT sham; #p<0.05, ##p<0.01, ###p<0.001 vs. WT cryo.

Category	Gene	Ad.Control	Ad.GATA4
Cell cycle promoting	Ccna2	1	1.2±0.16
	Ccnd1	1	1.02±0.1
	Ccnd2	1	1.35±0.26
	Ccnd3	1	0.94±0.12
	Ccne1	1	2.24±0.27**
	Cdk4	1	1.29±0.03***
	E2f1	1	1.15±0.15
	Cenpa	1	0.70±0.08*
	Zfp191	1	1.26±0.15
	Cdc2 (cdk1)	1	2.03±0.158***
Cell cycle inhibiting	Rbl1 (p107)	1	1.68±0.33
	Rab3gap1 (p130)	1	1.68±0.21*
	Tsc22d1	1	0.60±0.08**
Regenerative cytokines or receptors	Il13	1	1.41±0.1*
	Ctf1	1	2.53±0.23***
	Fgf16	1	0.4±0.03***
	Igf2r	1	1.47±0.09**

Appendix Table S2: Relative regenerative gene expression (average ± SEM) in neonatal rat cardiomyocytes treated with adenoviruses (Ad.) as indicated (n=6-9/group). *p<0.05, **p<0.01, ***p<0.001 vs. Ad.Control treated cells.

**Mouse Genes**	**Forward sequence 5' to 3'**	**Reverse sequence 5' to 3'**
Nppa	ATTGACAGGATTGGAGCCCAGAGT	TGACACACCACAAGGGCTTAGGAT
Nppb	CTCAAGCTGCTTTGGGCACAAGAT	AGCCAGGAGGTCTTCCTACAACAA
Gapdh	ACCCAGAAGACTGTGGATGG	CACATTGGGGGTAGGAACAC
Ccna2	CTTGGCTGCACCAACAGTAA	AGCAATGAGTGAAGGCAGGT
Ccnb1	TGGACTACGACATGGTGCAT	CTCCGTGTGGGACAGGTAGT
Ccnd1	CATTGCCCTTCCATCCTCTA	ACCAGCCTCTTCCTCCACTT
Ccnd2	TCGATGATTGCAACTGGAAG	ATGCTGCTCTTGACGGAACT
Ccnd3	TAGGCGCCTGCTCTATGTCT	ATCTGTGGGAGTGCTGGTCT
Ccne1	CACAACATCCAGACCCACAC	GGCAGGTTTGGTCATTCTGT
Cdk4	GTGCCAGAGATGGAGGAGTC	TTGTGCAGGTAGGAGTGCTG
E2F1	AGAGTGAGCAGCAGCTGGAT	CCATCTGTTCTGCAGGGTCT
Cenpa	ACACTGCGCAGAAGACAGAA	ACCACGGCTGAACTTCTCAC
Zfp91	CCTGGGAACTCCACTCTCTG	GCTGGGGAACACCTATGTTG
Cdc2 (cdk1)	AGCTCTGGGCACTCCTAACA	TCCATCCAGAGGGCTACATC
Rbl1 (P107)	TCCCACACATTAAGCAGCAG	CAGGTGAGTCTGCATCTCCA
Rab3gap1 (P130)	CTGGAGCAGCCTGAGGTATC	CTGGGCAGCCTGATTTCTTA
Tsc22d1	GCCTGGATTTCTCCGATTTT	TCACCGCATACATCAAATGG
Meis1	TCACACTGGCCTTAAAGAGGA	TGCCTACTCCATCCATACCC
Il13	TGCCAAGATCTGTGTCTCTCC	CCACACTCCATACCATGCTG
Ctf1	TCATTCCTACCCCATTTGGA	AGGGCTCTCCCTGTTGCT

Igf1	CACCCACAAAACAACACCTG	CACGAACTGAAGAGCATCCA
Igf2r	CGCAGGGCCAGAGTATAAAG	CCATCAGCCATTCTGTCTCA
Vegfa	CACGACAGAAGGAGAGCAGA	ACACAGGACGGCTTGAAGAT
Tgfb1	CACCATCCATGACATGAACC	TACTGTGTGTCCAGGCTCCA
Myh6	ACTGTGGTGCCTCGTTCC	GCCTCTAGGCGTTCCTTCTC
Myh7	AGCATTCTCCTGCTGTTTCC	CTCCAGCCTCTCCTTCTCAG
Kit	AAAGGCCAACATTCAAGCAG	GAGTTGACCCTCACGGAATG
Cd14	GCCTTTCTCGGAGCCTATCT	TGGCTTCGGATCTGAGAAGT
Vim	AGATCGATGTGGACGTTTCC	TCCGGTACTCGTTTGACTCC
Wt1	TGACTTCAAGGACTGCGAGA	CGCTGACAAGTTTTACACTGGA
Fgf16	GCTTCCACCTTGAGATCTTCC	TCTCCTCGCTCATTCATTCC
IL-13 promoter Primer 1	ACCCAGAACCTGGAAACCCT	GTGGCCGCTAAAGGAAAGAGT
IL-13 promoter Primer 2	CGTGCAGCTGAGGAACAATA	TTCCTCCCTTCCTTCCTTTC
**Rat Genes**	**Forward sequence 5' to 3'**	**Reverse sequence 5' to 3'**
Gapdh	ACCACCATGGAGAAGGCTGG	CTCAGTGTAGCCCAGGATGC
Ccna2	CTTGGCTGCACCAACAGTAA	AAGGCAGCTCCAGCAATAAG
Ccnd1	CACAACGCACTTTCTTTCCA	CAAGCCAGACCAGCTTCTTC
Ccnd2	CTTCAGCAGGACGAGGAAGT	GTGCTGCTCTTGACGGAACT
Ccnd3	CTTTGCGATGTACCCTCCAT	ATCTGTGGGAGTGCTGGTCT
Ccne1	GATGGGAAGTTCCAAGCTCA	GGCAGGTCTGGTCATTCTGT
Cdk4	GATTGCCTCCAGAAGACGAC	GGTCACTTTCCTCCTTGTGC

E2F1	CTGCAACAACTGCAGGAGAG	CCATTTGTTCTGCAGGGTCT
Cenpa	ACACTGCGCAGAAGACAGAA	ACCACGGCTGAACTTCTCAC
Zfp191	AGCTTGACGCCGTAGAGAAC	CTGAAGCGATCTCCTGCTTT
Cdc2 (cdk1)	CCCCTAACAACGAAGTGTGG	AGGGCCATTTTTCCAGAGAT
Rbl1 (P107)	GATGGGAAGTTCCAAGCTCA	GGCAGGTCTGGTCATTCTGT
Rab3gap1 (P130)	GCTGTCACTGAAGCGGAAAT	CCTGATACTGGGCCTGAGTC
Tsc22d1	CCTGGGCAATGACTTTGTTT	AGGGTCAACAGGTTGCATTT
Ctf1	TCATTCCTACCCCATTTGGA	AGGGCTCTCCCTGTTGCT
Igf2r	ACCAATTGCTGCAGAAGGAG	GCACCTGGATCTCTTCCATC
Il13	AGATCCACATCTCCCCCTGT	ATACCATGCTGCTGTTGCAC
Fgf16	GGGCTTCCACCTTGAAATCT	GATCCAAACAGCTCTCCTC
Raldh2	GAGAGTGACCCTGGAACTCG	GGCCCTTTCCACACTTCTTT
Tcf21	AATTGCTGCTACCTTCATCTTT	AGCTCACGGTTCCCAGACT
CD206	ATGCCAAGTGGGAAAATCTG	TGTAGCAGTGGCCTGCATAG
CD86	CAGTTACTGTGGCCCTCCTC	CAGCGTTACTATCCCGCTCT

Appendix Table S3: Sequences of primers used in quantitative real-time PCR.
