# Supplementary material for: The transcription factor GATA4 promotes myocardial regeneration in neonatal mice
Source: EMBO Mol Med. 2017 Jan 4;9(2):265–79. doi: 10.15252/emmm.201606602 (PMC5286367; doi:10.15252/emmm.201606602)
Supplement: Supplementary file 2 — Expanded View Figures PDF [file EMMM-9-265-s002.pdf]

Expanded View Figures

Figure EV1. GATA4 is necessary for cardiac regeneration after cryoinjury.

A Cardiac GATA4 protein abundance analyzed by immunoblotting in mice treated as indicated. + denotes positive control for GATA4 from cardiomyocytes infected with a GATA4-overexpressing adenovirus.

B Densitometric quantification of the immunoblot shown in (A); ***P = 0.0008 for sham vs. cryoinfarction and ***P < 0.0001 for 2 days (d) after sham surgery vs. 7 days after sham surgery.

C Quantification of left ventricular (LV) scar area of the indicated mice 7 days after cryoinjury (performed at P0).

D Cardiac GATA4 protein abundance analyzed by immunoblotting in mice treated as indicated.

E Quantification of the immunoblot shown in (D); **P = 0.0062.

F Quantification of left ventricular (LV) scar area of the indicated mice treated with adenoviruses (Ad.) as shown 7 days after cryoinjury; **P = 0.003 between Con and CM-G4-KO mice treated with control adenovirus (Ad.Con) and **P = 0.0043 between CM-G4-KO mice treated with Ad.Con or GATA4-overexpressing adenovirus (Ad.GATA4).

G Echocardiographic analysis of left ventricular systolic function in the indicated mice 2 months (M) after sham surgery or cryoinjury.

Data information: (B, C, E–G) The number within bars indicates the number of mice analyzed in that particular group. All data are expressed as mean ± SEM. Unpaired Student's t-test (C) and one-way ANOVA with Sidak's multiple comparisons test (B, E–G) were used to compare groups.

© 2017 The Authors

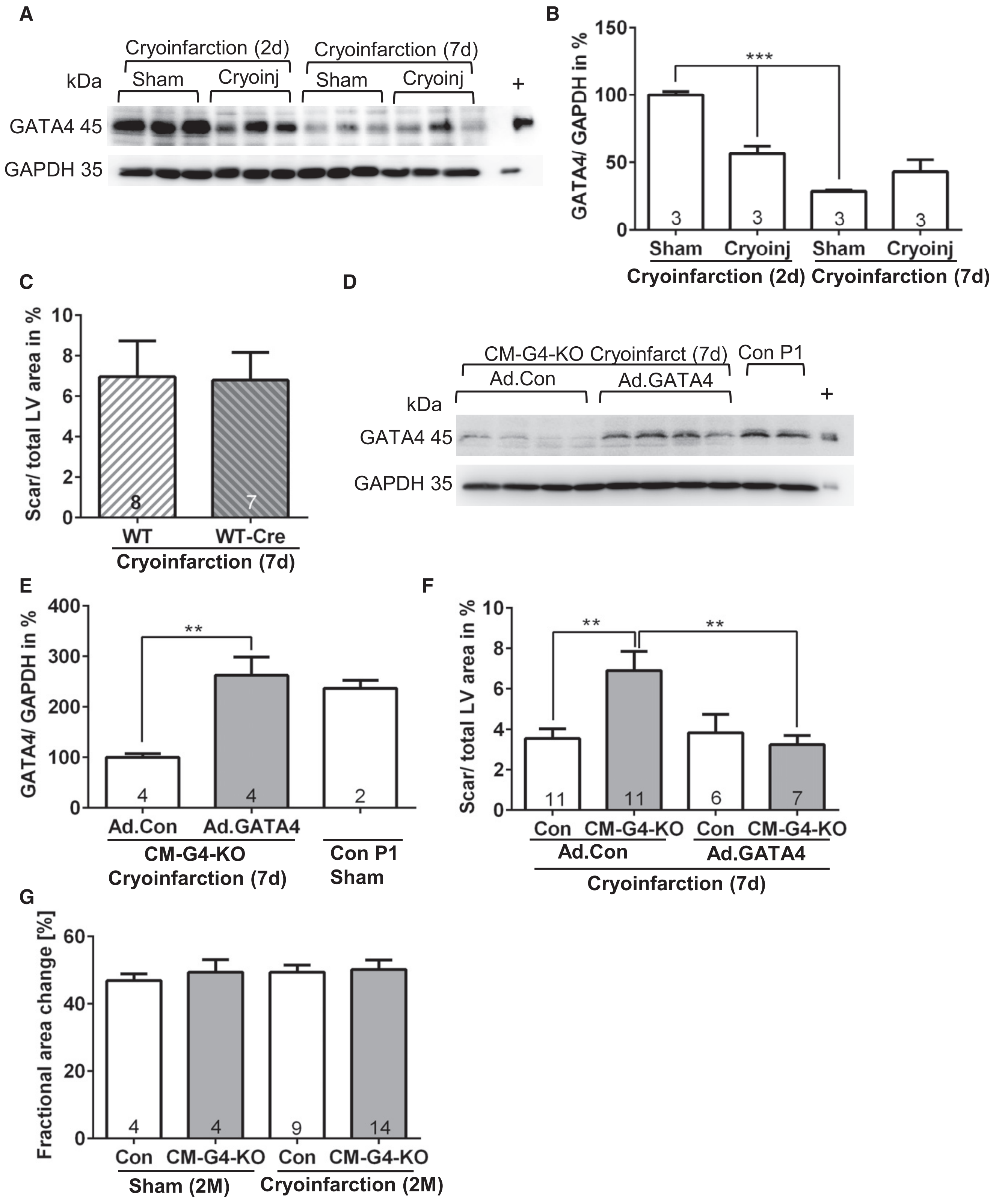
A
Cryoinfarction (2d)
Cryoinfarction (7d)
kDa
Sham
Cryoinj
Sham
Cryoinj
+
GATA4 45
GAPDH 35
B
GATA4/ GAPDH in %
***
Sham Cryoinj Sham Cryoinj
Cryoinfarction (2d) Cryoinfarction (7d)
C
Scar/ total LV area in %
WT
WT-Cre
Cryoinfarction (7d)
D
CM-G4-KO Cryoinfarct (7d)
Con P1
Ad.Con
Ad.GATA4
kDa
+
GATA4 45
GAPDH 35
E
GATA4/ GAPDH in %
**
Ad.Con
Ad.GATA4
CM-G4-KO
Cryoinfarction (7d)
Con P1
Sham
F
Scar/ total LV area in %
**
**
Con CM-G4-KO Con CM-G4-KO
Ad.Con
Ad.GATA4
Cryoinfarction (7d)
G
Fractional area change [%]
Con CM-G4-KO Con CM-G4-KO
Sham (2M)
Cryoinfarction (2M)

Figure EV1.

Figure EV2. Reduced angiogenesis and reduced cardiomyocyte proliferation in CM-G4-KO mice.

A Cardiac *Cd31* mRNA expression measured by qPCR in the indicated mice; ****$P < 0.0001$.
B Quantification of pH3-positive cardiomyocytes (CM) in mice 1 day (d) after sham or cryoinfarction as indicated; *$P = 0.011$.
C Quantification of cardiomyocyte cell division in mice 1 day (d) after sham or cryoinfarction as indicated; **$P = 0.0069$.
D Quantification of myocardial macrophage abundance (positive for F4/80) in the indicated mice 7 days after sham surgery or cryoinfarction. ***$P = 0.0003$.
E Heart weight/body weight (BW) ratio in the indicated mice 3 h after cryoinfarction.
F Heart weight/body weight ratio in the indicated mice 7 days after sham surgery or cryoinfarction.
G Representative wheat germ agglutinin (WGA)-stained myocardial sections for the quantification of cardiomyocyte (CM) cross-sectional area assessed 7 days after cryoinfarction in the indicated mice. Scale bars: 20 μm. The lower two pictures contain encircled cardiomyocyte cross sections (by a fine white line) of which the area was determined to obtain the cross-sectional area.
H qPCR-based determination of myocardial *Nppa* (known as ANP) and *Nppb* (BNP) mRNA expression as hypertrophic marker genes in mice as indicated.

Data information: The number within bars indicates the number of mice analyzed in that particular group. All data are expressed as mean ± SEM. Unpaired Student's *t*-test (E, G) and one-way ANOVA with Sidak's multiple comparisons test (A–D, F, H) were used to compare groups.

 © 2017 The Authors

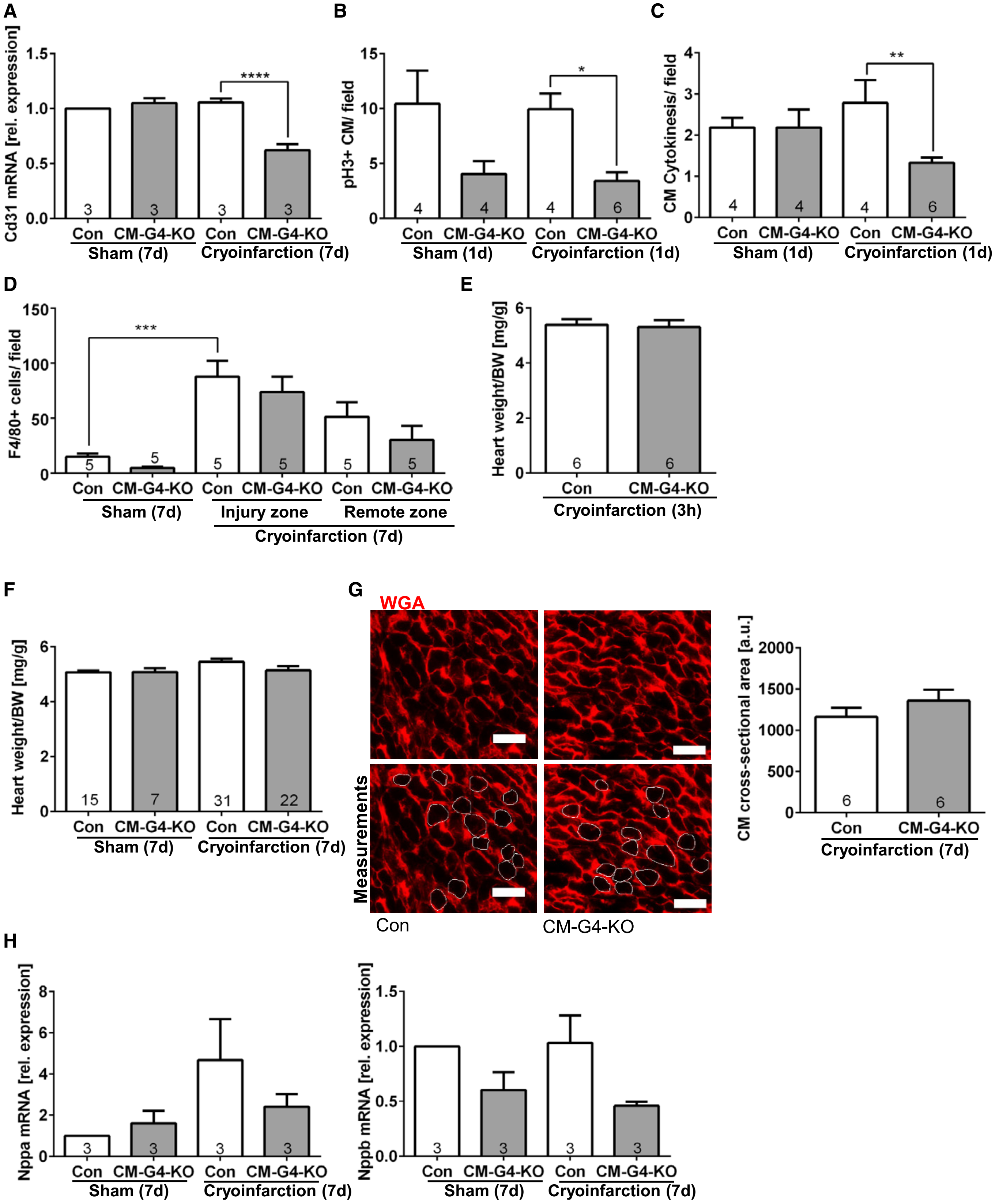
A
Cd31 mRNA [rel. expression]
****
Con CM-G4-KO Con CM-G4-KO
Sham (7d) Cryoinfarction (7d)
B
pH3+ CM/ field
*
Sham (1d) Cryoinfarction (1d)
C
CM Cytokinesis/ field
**
Sham (1d) Cryoinfarction (1d)
D
F4/80+ cells/ field
***
Sham (7d) Injury zone Remote zone
Cryoinfarction (7d)
E
Heart weight/BW [mg/g]
Cryoinfarction (3h)
F
Heart weight/BW [mg/g]
Sham (7d) Cryoinfarction (7d)
G
WGA
Measurements
Con
CM-G4-KO
CM cross-sectional area [a.u.]
Cryoinfarction (7d)
H
Nppa mRNA [rel. expression]
Nppb mRNA [rel. expression]
Sham (7d) Cryoinfarction (7d)

Figure EV2.

Figure EV3. Cell type-specific gene expression in CM-G4-KO mice.

A Expression of the indicated genes assessed by qPCR in the mice as shown; ****$P < 0.0001$. Data are expressed as mean ± SEM. One-way ANOVA with Sidak's multiple comparisons test was used to compare groups. Data are expressed as mean ± SEM. One-way ANOVA with Sidak´s multiple comparisons test was used to compare groups. The number within bars indicates the number of mice analyzed in that particular group.

B *In situ* hybridization to detect *Raldh2*, *Tcf21*, or *Tbx18* in the myocardium of the indicated mice 7 days after sham surgery or cryoinfarction to assess epicardial activation. Staining in the embryonic mouse heart served as positive (POS) control. Scale bar: 200 μm. A higher magnification of the epicardial region is shown on the right. Scale bar: 20 μm.

C c-kit immunofluorescence staining of a positive control (embryo liver lobes at embryonic day (E) 12) and 7-day-old heart tissues of the indicated mice after injury. Scale bars: 20 μm.

D Wt-1 immunofluorescence staining of 7-day-old Con and CM-G4-KO mice after cryoinjury. Scale bars: 200 μm.

E Myocardial immunofluorescence staining for CD3 as a marker of T cells of the indicated mice 7 days after cryoinjury. An embryonic heart section (from E12) was used as positive control. Scale bars: 20 μm.

© 2017 The Authors

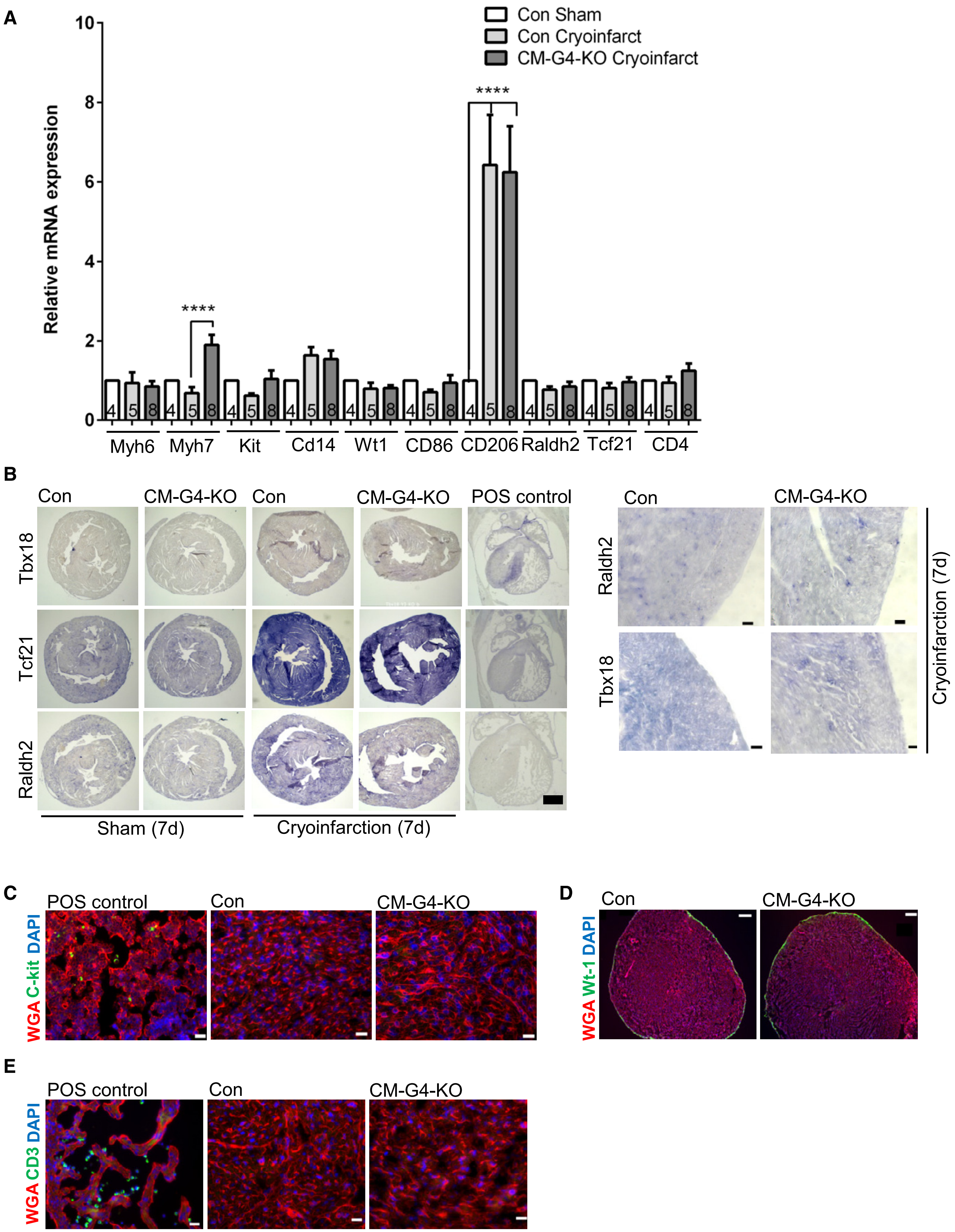
A
Con Sham
Con Cryoinfarct
CM-G4-KO Cryoinfarct
Relative mRNA expression
****
****
Myh6
Myh7
Kit
Cd14
Wt1
CD86
CD206
Raldh2
Tcf21
CD4
B
Con
CM-G4-KO
POS control
Tbx18
Tcf21
Raldh2
Sham (7d)
Cryoinfarction (7d)
C
WGA C-kit DAPI
D
WGA Wt-1 DAPI
E
WGA CD3 DAPI

Figure EV3.

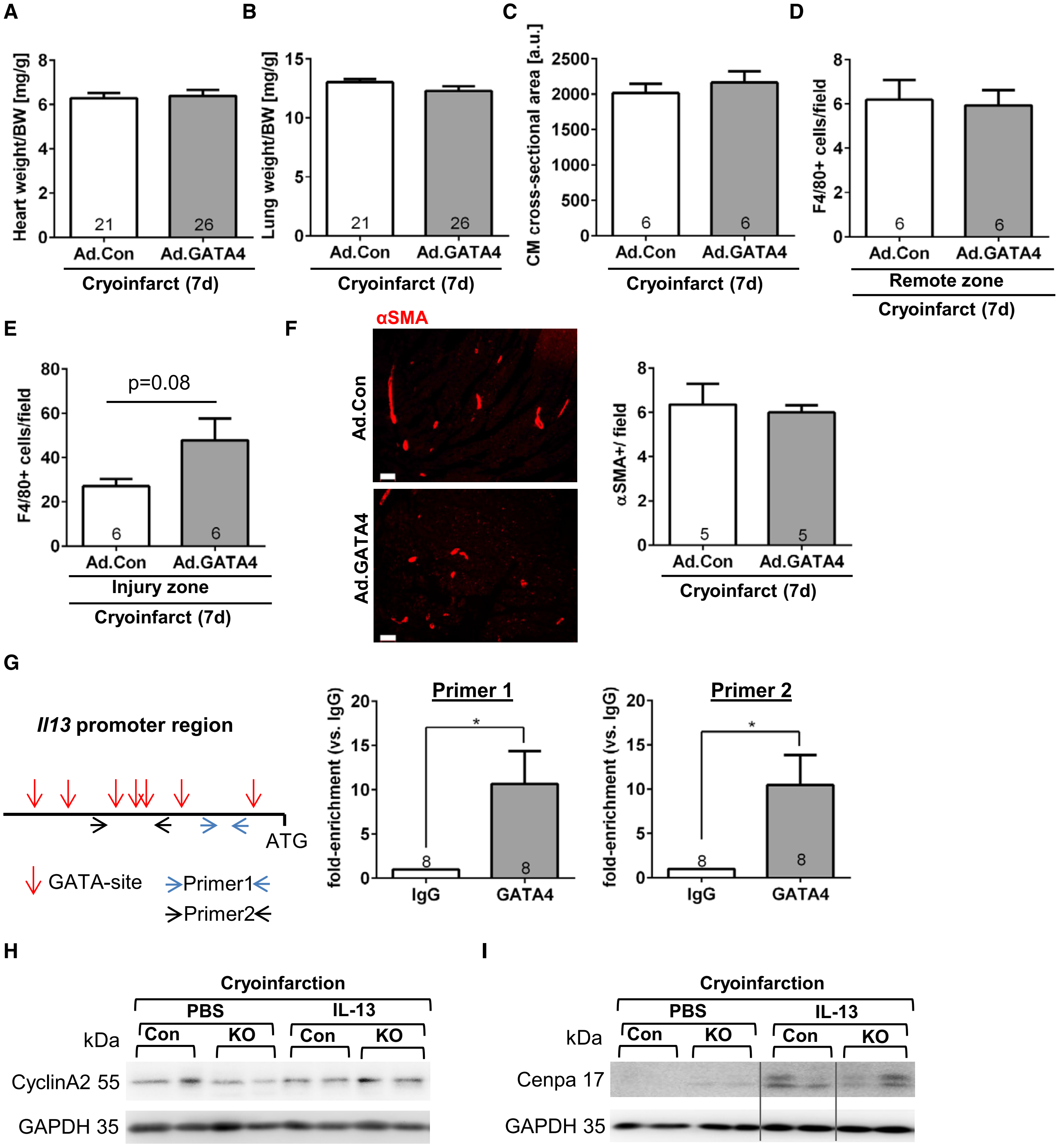

Figure EV4.

 © 2017 The Authors

Figure EV4. Additional characterization of GATA4 overexpression and IL-13 in neonatal hearts.

A Heart weight/body weight (BW) ratio of the indicated mice 7 days after sham surgery or cryoinfarction and application of adenoviruses as shown.
B Lung weight/body weight ratio of the indicated mice 7 days after sham surgery or cryoinfarction and application of adenoviruses as shown.
C Cardiomyocyte (CM) cross-sectional area assessed 7 days after cryoinfarction in the indicated mice.
D Quantification of myocardial macrophage abundance (positive for F4/80) remote of the infarcted area of the indicated mice.
E Quantification of myocardial macrophage abundance (positive for F4/80) within the infarcted area of the indicated mice.
F Myocardial immunofluorescence staining for αSMA in mice 7 days after cryoinjury and treatment with the indicated viruses. Quantification of small αSMA-positive small conductance vessel (~20–50 μm in diameter) in the myocardium of mice as shown. Scale bars: 50μm.
G Scheme of the mouse *Il13* promoter region (~700 bp) before the ATG translational start sites, GATA binding regions are marked by a red arrow, and the primers used are indicated by blue or black arrows. On the left, the results of a chromatin immunoprecipitation assay from neonatal hearts 7 days after cryoinjury and after immunoprecipitation with control IgG or GATA4 are shown. **P* = 0.0197 for primer 1 and **P* = 0.0142 for primer 2.
H Representative immunoblots for cyclin A2 and GAPDH from hearts of the mice as indicated (con = control, KO = CM-G4-KO).
I Representative immunoblots for cenpa and GAPDH from hearts of the mice as indicated. All lanes were run on the same gel but were non-contiguous where indicated by the gray lines.

Data information: (A–G) The number within bars indicates the number of mice analyzed in that particular group. All data are expressed as mean ± SEM. Unpaired Student's *t*-test was used to compare groups.
