# Supplementary figures and images for: The transcription factor GATA4 promotes myocardial regeneration in neonatal mice
Source: EMBO Mol Med. 2017 Jan 4;9(2):265–79. doi: 10.15252/emmm.201606602 (PMC5286367; doi:10.15252/emmm.201606602)

Figure 1A

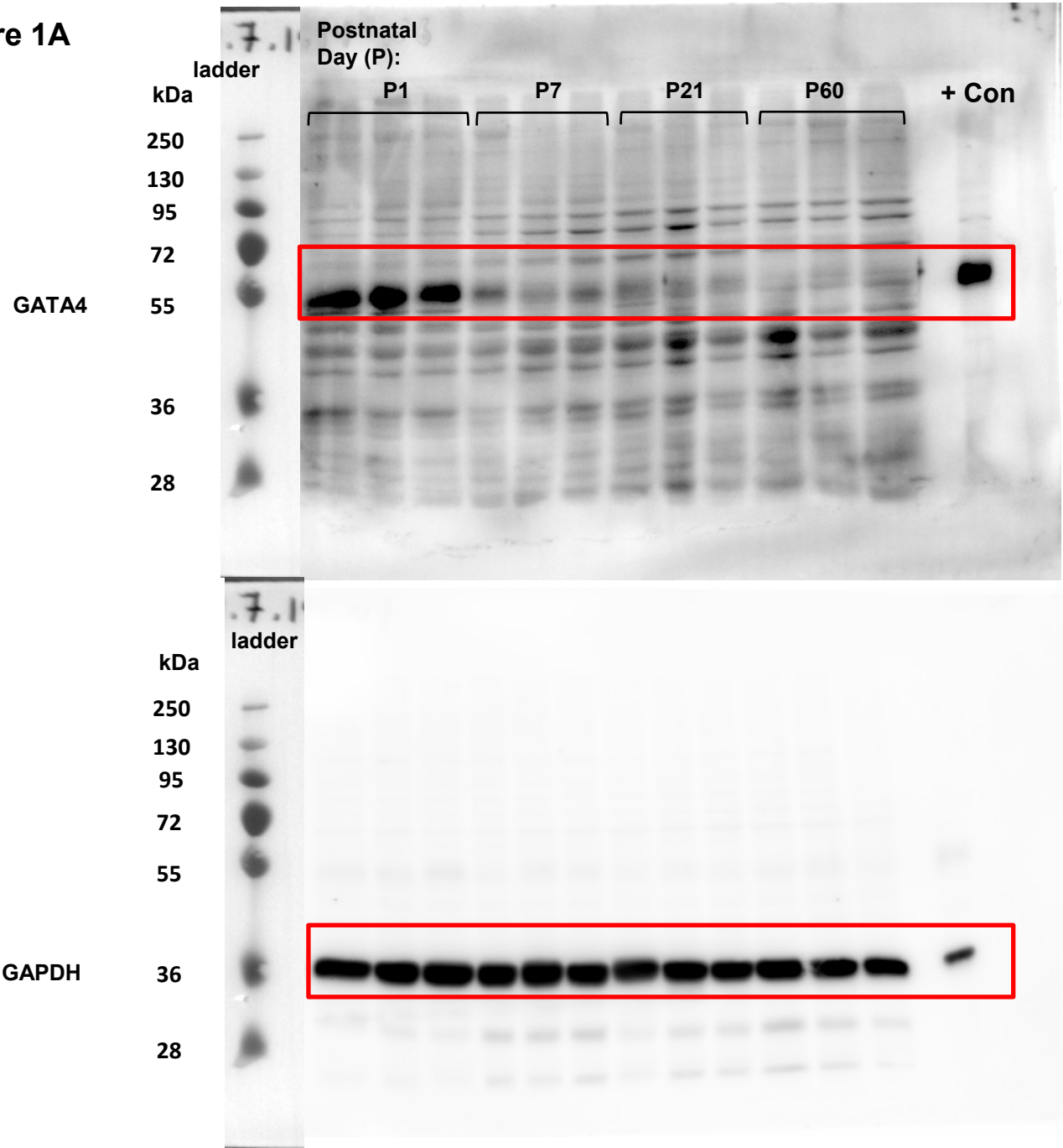

Figure 1C and Figure 3A

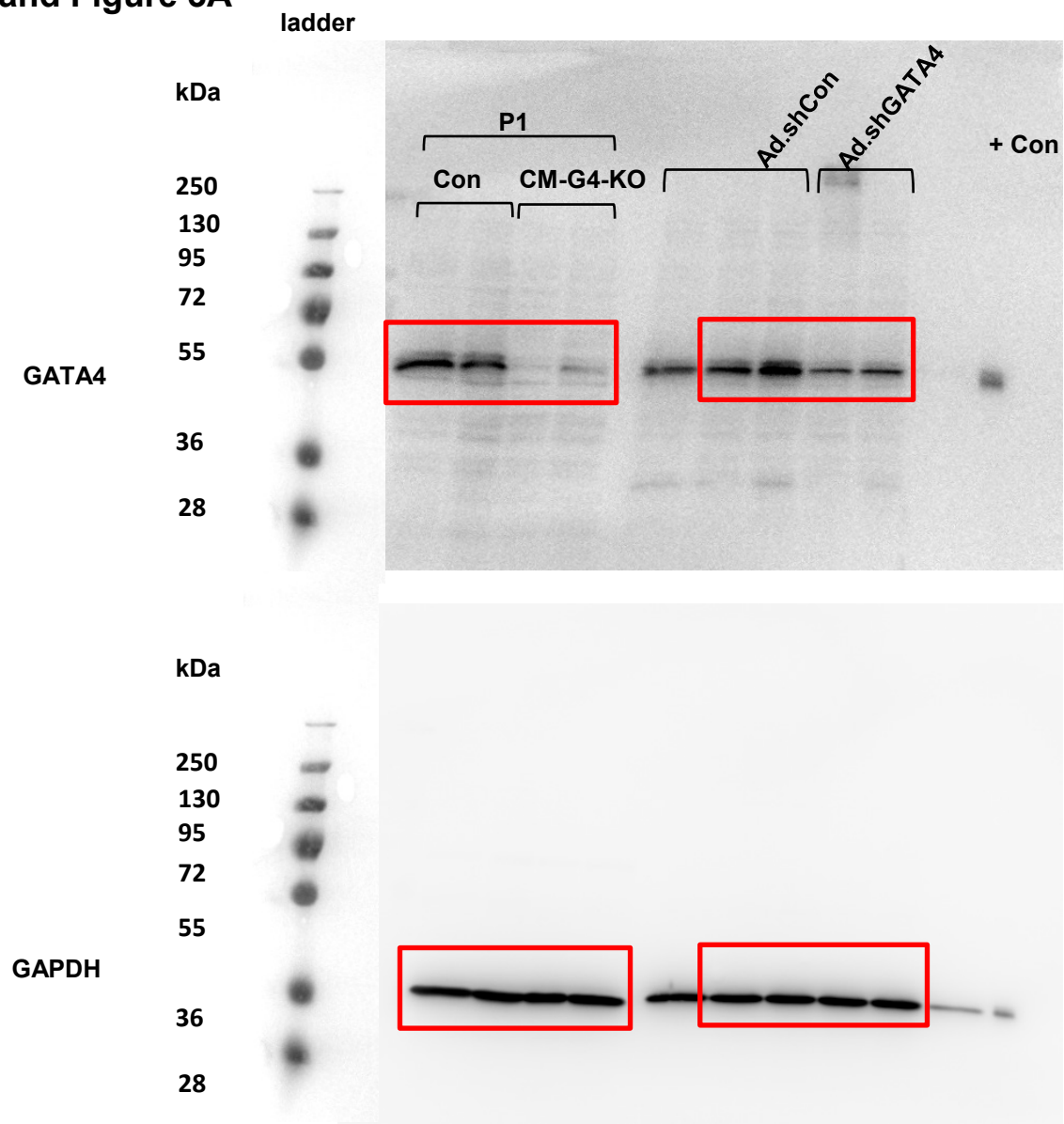

Supplement: Supplementary file 4 — Source Data for Figure 1 [file EMMM-9-265-s003.pdf]

Figure 1C and Figure 3A

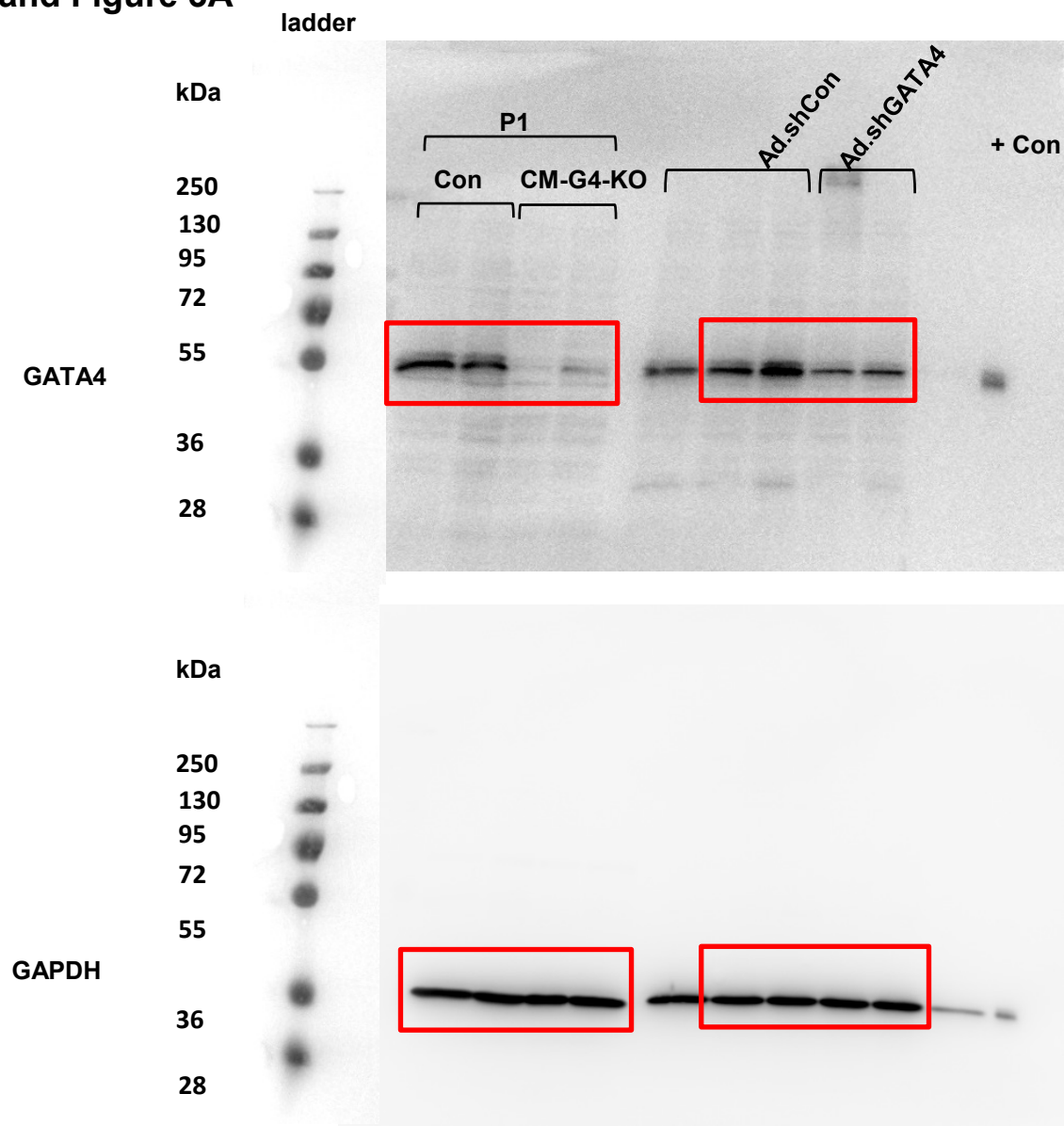

Supplement: Supplementary file 5 — Source Data for Figure 3 [file EMMM-9-265-s004.pdf]

Figure 4A

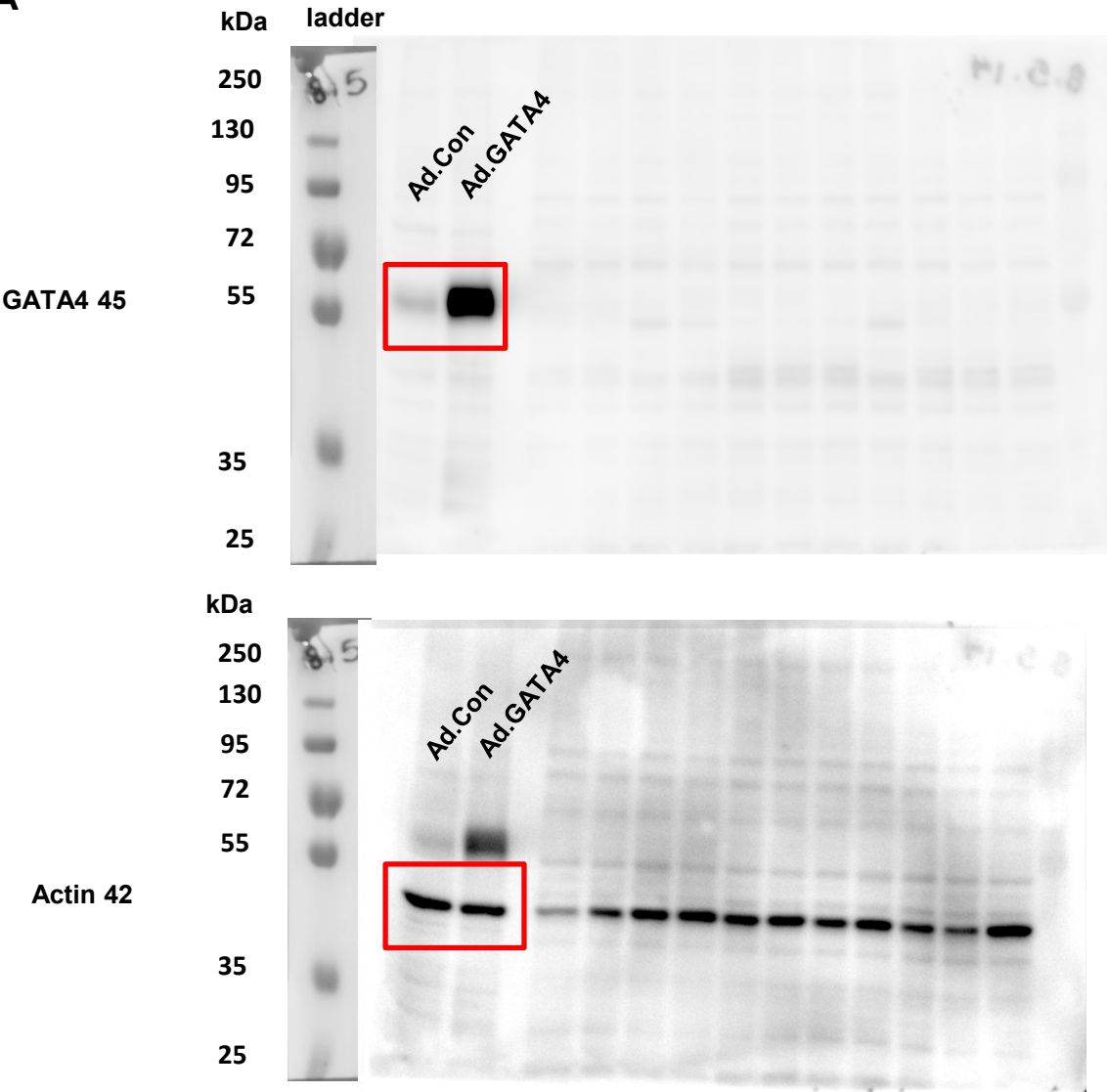

Figure 4C

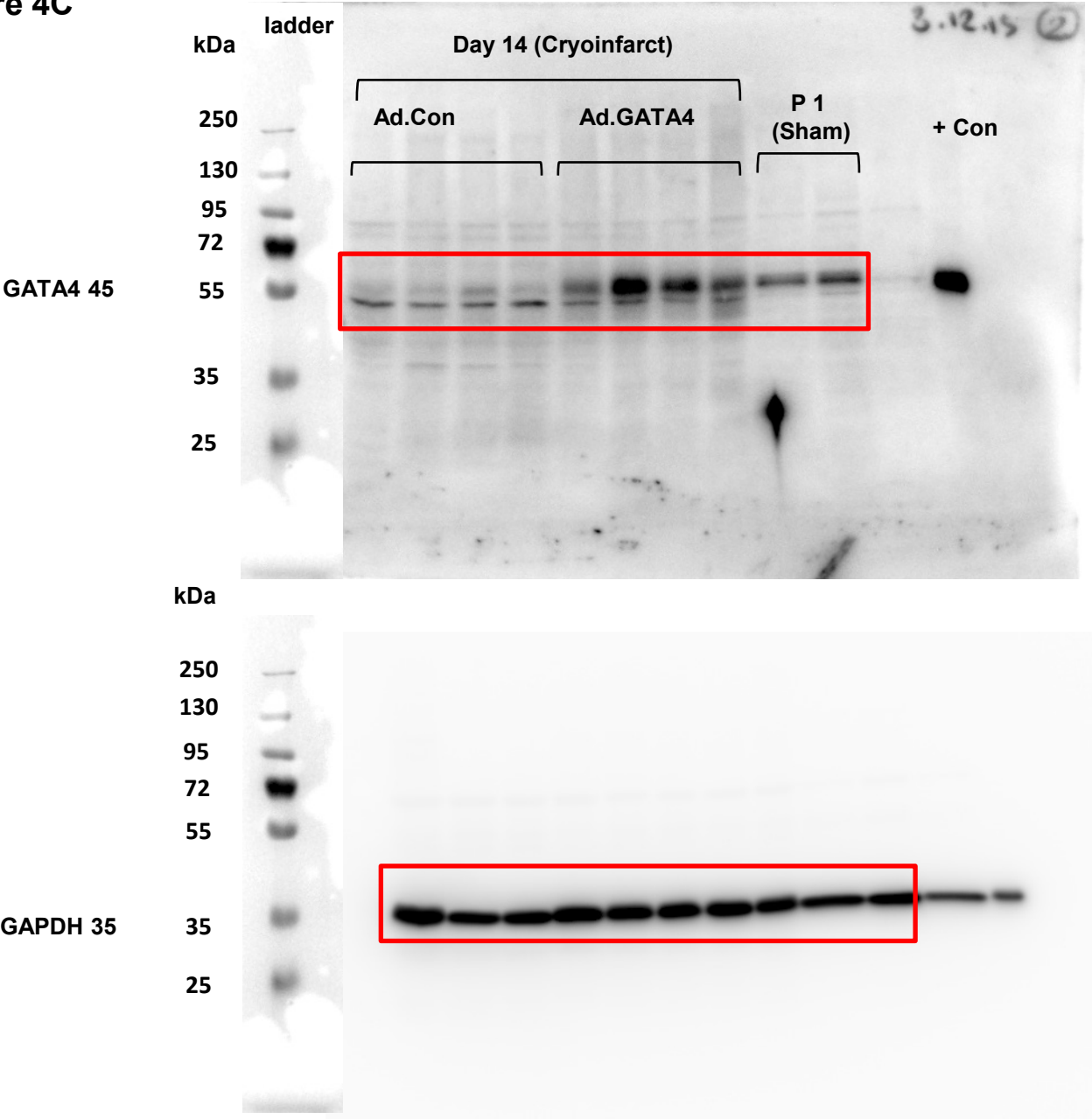

Supplement: Supplementary file 6 — Source Data for Figure 4 [file EMMM-9-265-s005.pdf]

Figure 5G

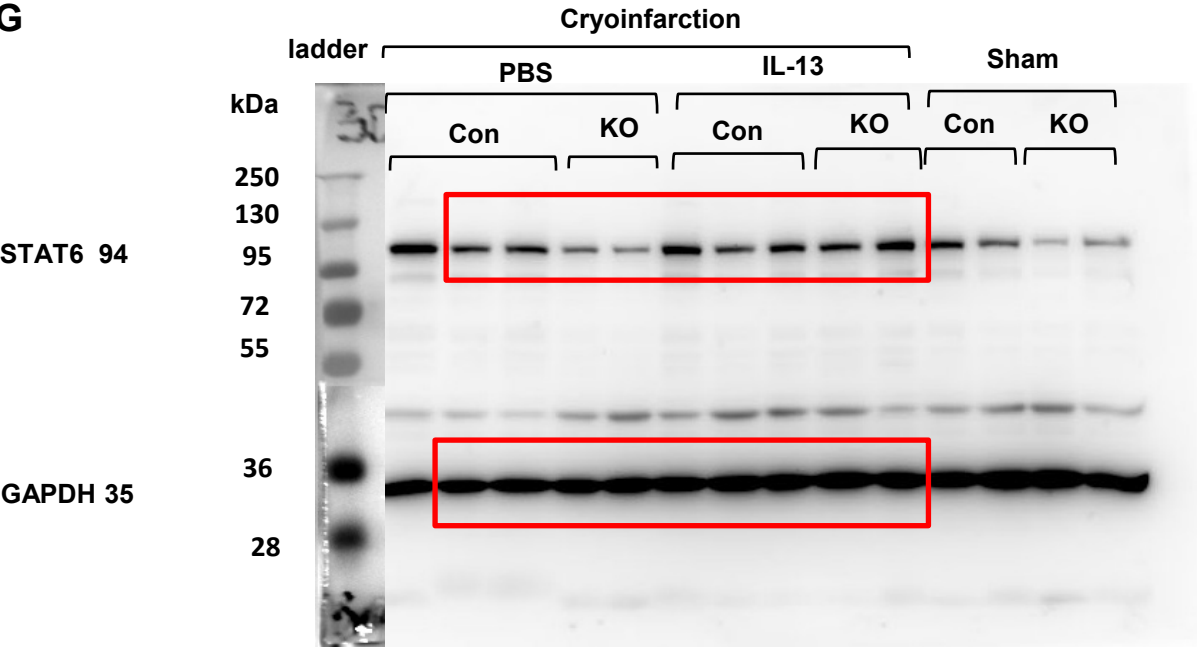

Supplement: Supplementary file 7 — Source Data for Figure 5 [file EMMM-9-265-s006.pdf]
